# Spatiotemporal Progression of Microcalcification in the Hippocampal CA1 Region following Transient Forebrain Ischemia in Rats: An Ultrastructural Study

**DOI:** 10.1371/journal.pone.0159229

**Published:** 2016-07-14

**Authors:** Tae-Ryong Riew, Yoo-Jin Shin, Hong Lim Kim, Jeong Min Cho, Ha-Jin Pak, Mun-Yong Lee

**Affiliations:** 1 Department of Anatomy, Catholic Neuroscience Institute, Cell Death Disease Research Center, College of Medicine, The Catholic University of Korea, 137-701, Seoul, Korea; 2 Integrative Research Support Center, Laboratory of Electron Microscope, College of Medicine, The Catholic University of Korea, Seoul, Korea, 137-701, Seoul, Korea; Federico II University of Naples, ITALY

## Abstract

Calcification in areas of neuronal degeneration is a common finding in several neuropathological disorders including ischemic insults. Here, we performed a detailed examination of the onset and spatiotemporal profile of calcification in the CA1 region of the hippocampus, where neuronal death has been observed after transient forebrain ischemia. Histopathological examinations showed very little alizarin red staining in the CA1 pyramidal cell layer until day 28 after reperfusion, while prominent alizarin red staining was detected in CA1 dendritic subfields, particularly in the stratum radiatum, by 14 days after reperfusion. Electron microscopy using the osmium/potassium dichromate method and electron probe microanalysis revealed selective calcium deposits within the mitochondria of degenerating dendrites at as early as 7 days after reperfusion, with subsequent complete mineralization occurring throughout the dendrites, which then coalesced to form larger mineral conglomerates with the adjacent calcifying neurites by 14 days after reperfusion. Large calcifying deposits were frequently observed at 28 days after reperfusion, when they were closely associated with or completely engulfed by astrocytes. In contrast, no prominent calcification was observed in the somata of CA1 pyramidal neurons showing the characteristic features of necrotic cell death after ischemia, although what appeared to be calcified mitochondria were noted in some degenerated neurons that became dark and condensed. Thus, our data indicate that intrahippocampal calcification after ischemic insults initially occurs within the mitochondria of degenerating dendrites, which leads to the extensive calcification that is associated with ischemic injuries. These findings suggest that in degenerating neurons, the calcified mitochondria in the dendrites, rather than in the somata, may serve as the nidus for further calcium precipitation in the ischemic hippocampus.

## Introduction

Brain parenchymal calcification is a well-known finding in various kinds of neuropathological disorders including brain ischemia [1–9]. These previous studies revealed that calcium precipitation is associated with neurodegeneration and impaired calcium homeostasis. Degenerating neurons frequently exhibit intracellular calcium aggregates and are regarded as the primary source for calcium deposits in the brain [7, 10–13]. Calcification development in areas of neuronal degeneration depends on the extent and nature of the injury, the age of the animals, and the brain regions affected; further, the heterogeneity of the astroglial population contributes to the variations that occur in the regional vulnerability to neurodegeneration and calcium deposition [7, 8, 14, 15]. Although the pathogenic mechanisms of these calcifications are not fully understood, it is now widely accepted that the formation of insoluble calcium phosphate complexes reduces free cytoplasmic calcium ions in neurons, which can be viewed as a potential compensatory mechanism for reducing further brain damage [6, 7, 10, 12, 16].

Transient forebrain ischemia for 10 min causes the selective and delayed neuronal death of hippocampal CA1 neurons in the rat brain [17–19]. Calcium precipitates can be detected in the swollen and disrupted mitochondria of these selectively vulnerable neurons at very early time points (within 6 h) after ischemic injury [4, 20, 21]. In addition, Maetzler, Stunitz (10) demonstrated that neuronal mitochondria act as candidate structures for calcium deposit nucleation. These data suggest that intracellular calcification initially occurs in the mitochondria of degenerating neurons due to an excess of calcium, and this in turn disrupts the structural and functional integrity of the organelles. However, detailed knowledge of the cellular events that occur within the degenerated cells prior to, and following, the onset of mineralization has not yet been obtained, despite the fact that such knowledge is central to understanding the calcification process within the brain.

Therefore, we performed a detailed ultrastructural study of the onset and spatiotemporal progression of calcification in the CA1 region of the hippocampus during a 4-week survival period after transient forebrain ischemia. We used several different methods including conventional transmission electron microscopy (TEM), field emission (FE)-TEM with electron probe microanalysis, and alizarin red S staining for light microscopy, which is a sensitive and specific method for detecting calcium deposition [13, 22]. To detect calcium precipitation, we used the osmium/potassium-bichormate method to precipitate and ultrastructurally visualize endogenous calcium [23]. We focused our attention on the temporal pattern of calcification that occurs 3 to 14 days after reperfusion because extensive cell death has been observed within 2 to 3 days after ischemia and reaches its maximal effect within 1–2 weeks [17–19]. Our results provide new information that links intracellular calcium precipitation to the extracellular calcification process that occurs in the ischemic brain.

## Materials and Methods

### Animal preparation

All experimental procedures were conducted in accordance with the Laboratory Animal Welfare Act, Guide for the Care and Use of Laboratory Animals, and Guidelines and Policies for Rodent Survival Surgery, and were approved by the Institutional Animal Care and Use Committee at the College of Medicine of The Catholic University of Korea (Approval Number: CUMC-2014-0006-01). All efforts were made to minimize animal suffering and to reduce the number of animals used.

Adult male Sprague Dawley rats (250–300 g) were used in this study. Animals were housed in groups of three per cage in a controlled environment at a constant temperature (22 ± 5°C) and humidity (50 ± 10%) with food (gamma ray-sterilized diet) and water (autoclaved tap water) available *ad libitum*. They were maintained on a 12-h light/dark cycle. Transient forebrain ischemia was induced by the four-vessel occlusion and reperfusion method described by Pulsinelli and Brierley [24], with minor modifications [25]. Briefly, the vertebral arteries were electrocauterized and cut to stop circulation in these vessels. After 24 h, both common carotid arteries were occluded for 10 min with miniature aneurismal clips. Only those animals lacking a righting reflex after vascular occlusion were classified as ischemic and used in the study. Body temperatures (measured rectally) were maintained at 37.5 ± 0.3°C with a heating lamp during and after ischemia. Sham-operated rats, with cauterized vertebral arteries and ligatures placed around the carotid arteries, were used as controls. No animal convulsed or died following reperfusion or sham operation. After the surgery, animals were monitored twice daily to determine their health and activity levels, i.e., we examined their behavioral changes (activity and food intake), body weight, and body temperature. The overall mortality rate of ischemic rats was lower than 5%, and all sham-operated rats survived after the operation. Most of the deaths occurred during or shortly after the ischemic procedure, and the leading cause of death appeared to be related to cardiac arrest. However, none of the rats were eliminated from the analysis in the days following the ischemic insult owing to sickness or death, and we did not observe any prominent changes in the behavior or body weight of the rats during the experiments.

### Ultrastructural analysis and mineral characterization

Animals were allowed to live for 3, 7, 14, or 28 days after reperfusion. At each of the four time points following reperfusion, animals (*n* = 8 per time point for the ischemic group; *n* = 5 per time point for the sham-operated group) were deeply anesthetized with 16.9% urethane (10 mL/kg via intraperitoneal injection) and sacrificed by transcardial perfusion with a fixative containing 4% paraformaldehyde in 0.1 M phosphate buffer (pH 7.4). The brains ware promptly removed, sectioned with a vibratome at 40 μm, and post-fixed with 1% osmium tetroxide (OsO_4_) and 2.5% potassium-dichromate (K_2_Cr_2_O_7_; Hayashi Pure Chemical Industries Ltd., Osaka, Japan) for 30 min at room temperature (25°C). After dehydration and embedding in Epon 812, areas of interest were excised and glued onto resin blocks. Ultrathin sections (70–90-nm thick) were lightly stained with uranyl acetate and observed with an electron microscope (JEM-1010; JEOL, Tokyo, Japan).

For the electron probe x-ray microanalysis, ultrathin sections were placed on copper grids and carbon coated (at a thickness of approximately 20 nm). The chemical compositions of the selected profiles were analyzed using FE-TEM (JEM-2100F, JEOL). The microscope was equipped with Energy Dispersive X-ray Analysis (EDAX), which was installed at the Korea Basic Science Institute (Daejeon, Korea). The operative voltage was 200 kV.

### Histological study

To define the relationship between ectopic calcium deposition and cell death, we employed the alizarin red S and Fluoro-Jade B (FJB) histochemical techniques. For this experiment, we used additional groups of rats, including sham controls and experimental rats at 3, 7, 14, or 28 days after reperfusion (*n* = 3 per group). Sequential coronal cryostat sections (25-μm thick) were stained with 1% (w/v) alizarin red S (SIGMA, St. Louis, MO, USA) for 6 min or with 0.0004% FJB (Millipore, Billerica, MA, USA) in distilled water containing 0.01% acetic acid for 30 min according to the manufacturer’s protocol. After rinsing in distilled water, the sections were immersed in xylene and cover-slipped with the mounting medium. Sections stained with alizarin red were viewed with a microscope and photographed with a digital camera (Jenoptik, Germany). Images were converted to the tagged image file format, and the contrast levels were adjusted using Adobe Photoshop v. 10.0 (Adobe Systems, San Jose, CA, USA). Since the images were acquired with the same light intensity during microscopy and the same parameters for digital photography, only minor adjustments were made to establish uniform contrast settings for all figures. Slides stained with FJB were viewed with a confocal microscope (LSM 510 Meta; Carl Zeiss Co., Ltd., Oberkochen, Germany).

## Results

### Spatiotemporal relationship between neuronal cell death and calcium deposits in the CA1 region of the ischemic hippocampus

**Fig 1:**
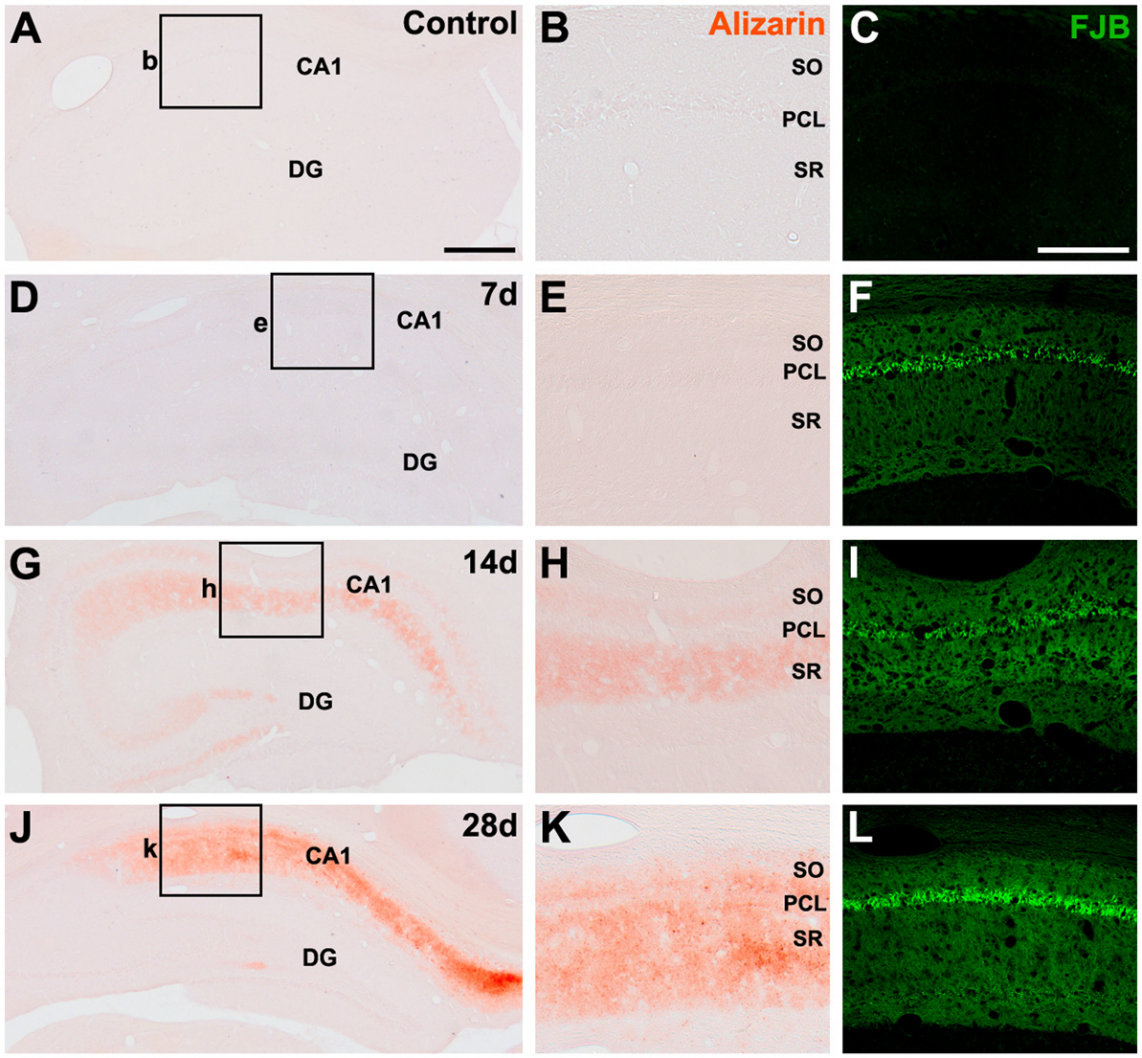
Spatial and temporal relationships between alizarin red and Fluoro-Jade B (FJB) staining in the hippocampal CA1 region in sham-operated rats (A–C) and rats exposed to ischemic insult at 7 (D–F), 14 (G–I), and 28 days (J–L) after reperfusion. (A–C) No significant alizarin red or FJB staining was detected in the hippocampus of control animals. (D–F) At 7 days after reperfusion, alizarin red staining was negligible in the CA1 region of the hippocampus, while FJB staining was more intense in the pyramidal cell layer (PCL) than it was in the dendritic subfields including the strata oriens (SO) and radiatum (SR). (G–I) At 14 days after reperfusion, amorphous alizarin red staining was observed over the CA1 dendritic subfields but not in the PCL. Note that intense FJB staining was still observed in the PCL. (J–L) At 28 days after reperfusion, the amorphous to granule-like alizarin red staining was still restricted to within the CA1 dendritic strata, while intense FJB staining was noted in the CA1 PCL. Scale bars = 500 μm for A, D, G, J; 250 μm for B, C, E, F, H, I, K, L.

First, we examined whether ectopic calcium deposition occurs in association with neuronal cell death in hippocampi subjected to 10-min ischemic insults. To do this, we applied the alizarin red S technique and FJB, a fluorescent dye that labels degenerating neurons, to serial hippocampal sections at different time points after reperfusion. No significant staining for either alizarin red or FJB was detected in sham-operated animals (Fig 1A–1C). At 3 (data not shown) and 7 days (Fig 1D–1F) after reperfusion, alizarin red staining was negligible in the hippocampus, while FJB-stained sections showed selective neurodegeneration in the CA1 region of the hippocampus. In CA1, FJB staining was more intense in the pyramidal cell layer than it was in the dendritic subfields including the stratum oriens and radiatum (Fig 1F). At days 14 (Fig 1I) and 28 (Fig 1L) after reperfusion, the patterns of FJB staining were similar to those at day 7. In contrast, amorphous alizarin red staining was evident in the CA1 dendritic layers, but not in the pyramidal cell layer, by 14 days after reperfusion (Fig 1G and 1H). This alizarin red staining remained unchanged until 28 days after reperfusion; however, increased staining was clearly evident in the stratum radiatum and oriens, where granule-like alizarin red staining was occasionally detected (Fig 1J and 1K).

### Electron microscopic analysis of the CA1 hippocampal area of reperfused rats at days 3 and 7 after transient forebrain ischemia

**Fig 2:**
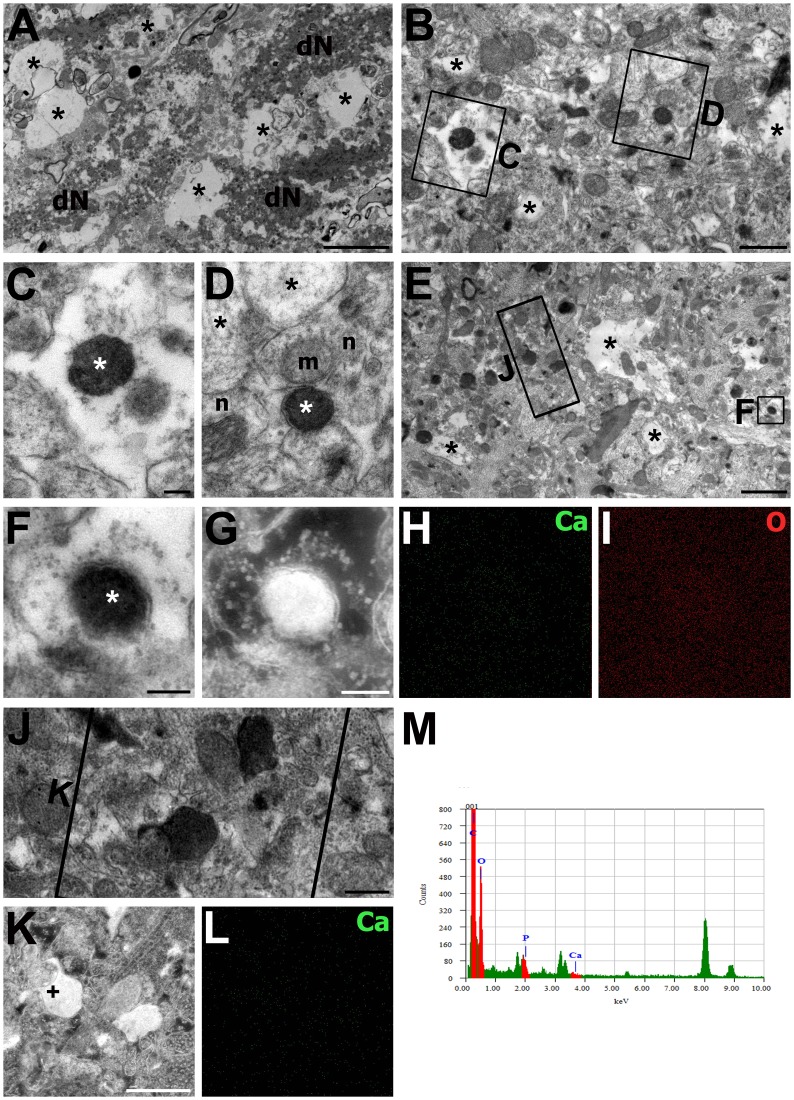
Electron photomicrographs of the CA1 region of the hippocampus at 3 days after reperfusion. **Images were acquired using the osmium/potassium dichromate method.** (A, B, E) Lower magnification views of the CA1 pyramidal cell layer (A) and the stratum radiatum (B, E). (A) Large electron-lucid vacuoles (asterisks) and dark and contracted perikarya (dN) with unrecognizable cell organelles were frequently observed. (B, E) The stratum radiatum exhibited alterations in the arrangement and morphology of neurites and displayed vacuoles of different sizes (asterisks) caused by the swollen neurites. (C, D) Higher magnification views of the boxed areas in B. Electron-dense mitochondria (white asterisks), which can be identified by their recognizable cristae, were observed within the swollen dendrites (C) or within the relatively intact neurites (D) that abutted the neurites (n in D) containing normal-appearing mitochondria (m in D). Black asterisks in D denote swollen neurites. (F, J) Higher-magnification views of the boxed areas in E. (F–L) Bright-field TEM micrographs (F, J) and associated (G, K), calcium (H, L) and oxygen (I) FE-TEM maps showing the absence of calcium in an electron-dense mitochondrial profile (white asterisk in F). (M) X-ray spectrum analysis of the area marked by the cross in (K) showing an absence of calcium (Ca) peaks. Scale bars = 4 μm for A; 2 μm for E; 1 μm for B; 0.5 μm for J; 0.4 μm for K, L; 0.25 μm for G–I; 0.2 μm for C, D, F.

Next, we performed a comparative ultrastructural study of the CA1 region of the hippocampus using the osmium/potassium dichromate method. At 3 days after reperfusion (Fig 2A), most of the CA1 pyramidal neurons revealed classical necrotic cell death characterized by cytoplasmic swelling and the eventual rupture of nuclear and plasma membranes, which were replaced by large vacuoles containing amorphous remnants [26, 27]. In addition, some dark degenerating neurons were scattered among these vacuoles throughout the CA1 pyramidal cell layer; these neurons had markedly contracted perikarya with pronounced disintegration of the cytoplasmic organelles as well as nuclei that were no longer discernible (Fig 2A). In the stratum radiatum, which showed alterations in the arrangement and morphology of neurites, swollen dendrites containing electron-dense mitochondria were frequently observed, some of which were completely filled with electron-dense materials and had no recognizable internal cristae (Fig 2B, 2C, 2E and 2F). Electron-dense mitochondria were also found within relatively intact neurites that touched the adjacent neurites with normal-appearing mitochondria (Fig 2B, 2D, 2E and 2J). Then, we identified whether the increased electron density within these mitochondria was due to calcium precipitates. FE-TEM with EDAX revealed no prominent signals or peaks for calcium within the electron-dense mitochondria of degenerating neurites (Fig 2F–2M).

**Fig 3:**
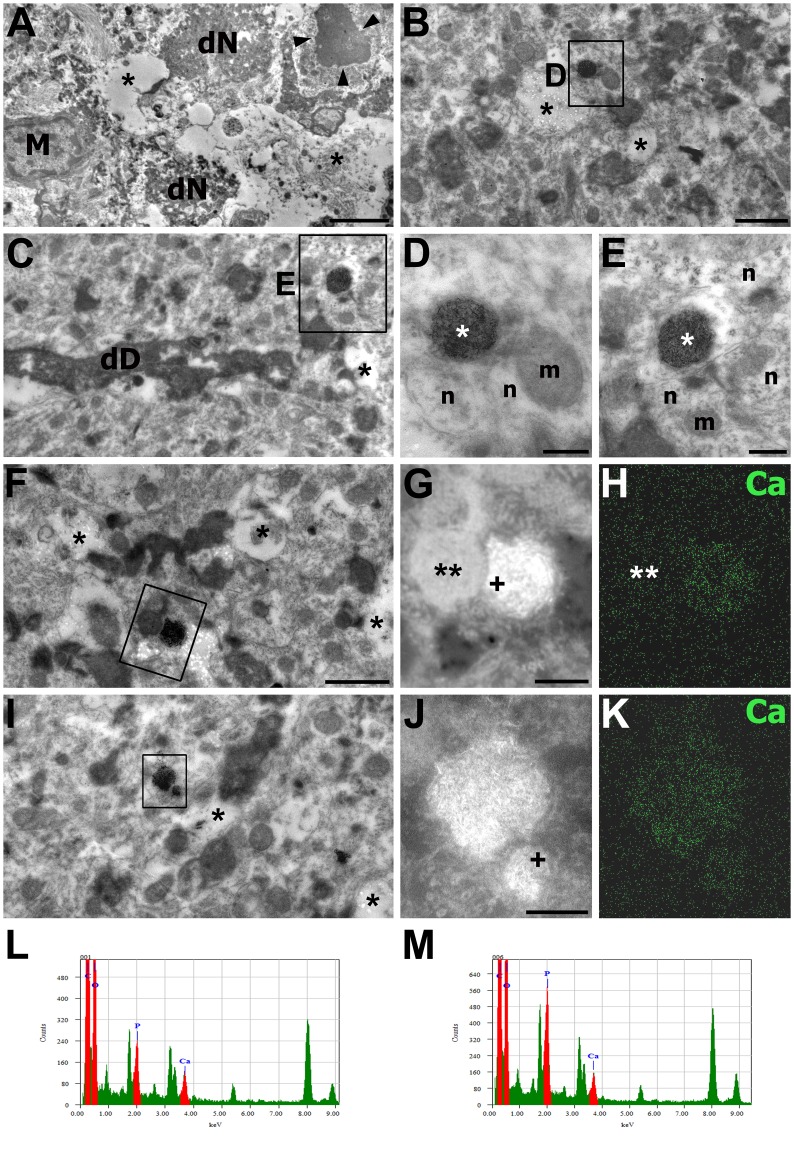
Electron photomicrographs of the CA1 hippocampal area at 7 days after reperfusion. The images were acquired using the osmium/potassium dichromate method. (A) Lower magnification views of the CA1 pyramidal cell layer. Dark neuronal fragments (dN), neuronal remnants containing chromatin clumps (arrowheads) resembling apoptotic bodies, large electron-lucid vacuoles (asterisks), and microglial cells (M) were frequently observed in this layer. (B, C, F, I) Lower magnification views of the CA1 stratum radiatum. Darkened, degenerated profiles originating from dendritic shafts (dD in C) and distal dendrites and vacuoles of different sizes (asterisks) were observed in this stratum. (D, E) Higher-magnification views of the boxed areas in B and C, respectively. Degenerated dendrites contained electron-dense mitochondria (white asterisks) that were filled with small, needle-like calcium precipitates, and were often directly apposed to neurites (n) containing normal-appearing mitochondria (m). (G, H, J, K) Dark-field images (G, J) and calcium maps (H, K) corresponding to the TEM images shown in F and I, respectively. Note that calcium-related signals were conspicuous in mitochondria filled with needle-like crystals but not in mitochondria located in the adjacent neurites (double asterisks in G, H). (L, M) X-ray spectrum analysis obtained from the selected points (crosses) in G and J, respectively. Note the significant peaks for calcium (Ca) and phosphorous (P) inside these mitochondria. Scale bars = 4 μm for A; 1 μm for B, C, F, I; 0.3 μm for G, H; 0.2 μm for D, E, J, K.

At 7 days after reperfusion, large electron-lucid vacuoles and darkly stained neuronal fragments were still observed in the CA1 pyramidal cell layer (Fig 3A). In addition, microglial cells and neuronal remnants containing chromatin clumps that resembled apoptotic bodies were observed in this layer. The ultrastructural features of the stratum radiatum at 7 days after reperfusion were similar to those at 3 days except that degenerating neurites were more frequently observed (Fig 3B, 3C, 3F and 3I) and the dendrites, including the dendritic shafts, became homogenized and more electron-dense at 7 days after reperfusion (Fig 3C). Some degenerating dendrites had electron-dense mitochondria filled with small, needle-like precipitates that were compatible with calcium deposits (Fig 3D and 3E). These dendrites were often in direct proximity to what appeared to be normal neurites (Fig 3D and 3E) or to other neurites containing electron-dense mitochondria (Fig 3F and 3I). FE-TEM with electron probe microanalysis revealed conspicuous calcium-related signals in these electron-dense mitochondria, as expected (Fig 3F–3K), but such signals were not observed in the apparently normal mitochondria located in the adjacent neurites (Fig 3F–3H). In addition, X-ray spectrum analysis obtained from representative regions inside these calcifying mitochondria revealed significant peaks for both calcium and phosphorous (Fig 3L and 3M).

### Electron microscopic analysis of the CA1 hippocampal area of reperfused rats at days 14 and 28 after transient forebrain ischemia

**Fig 4:**
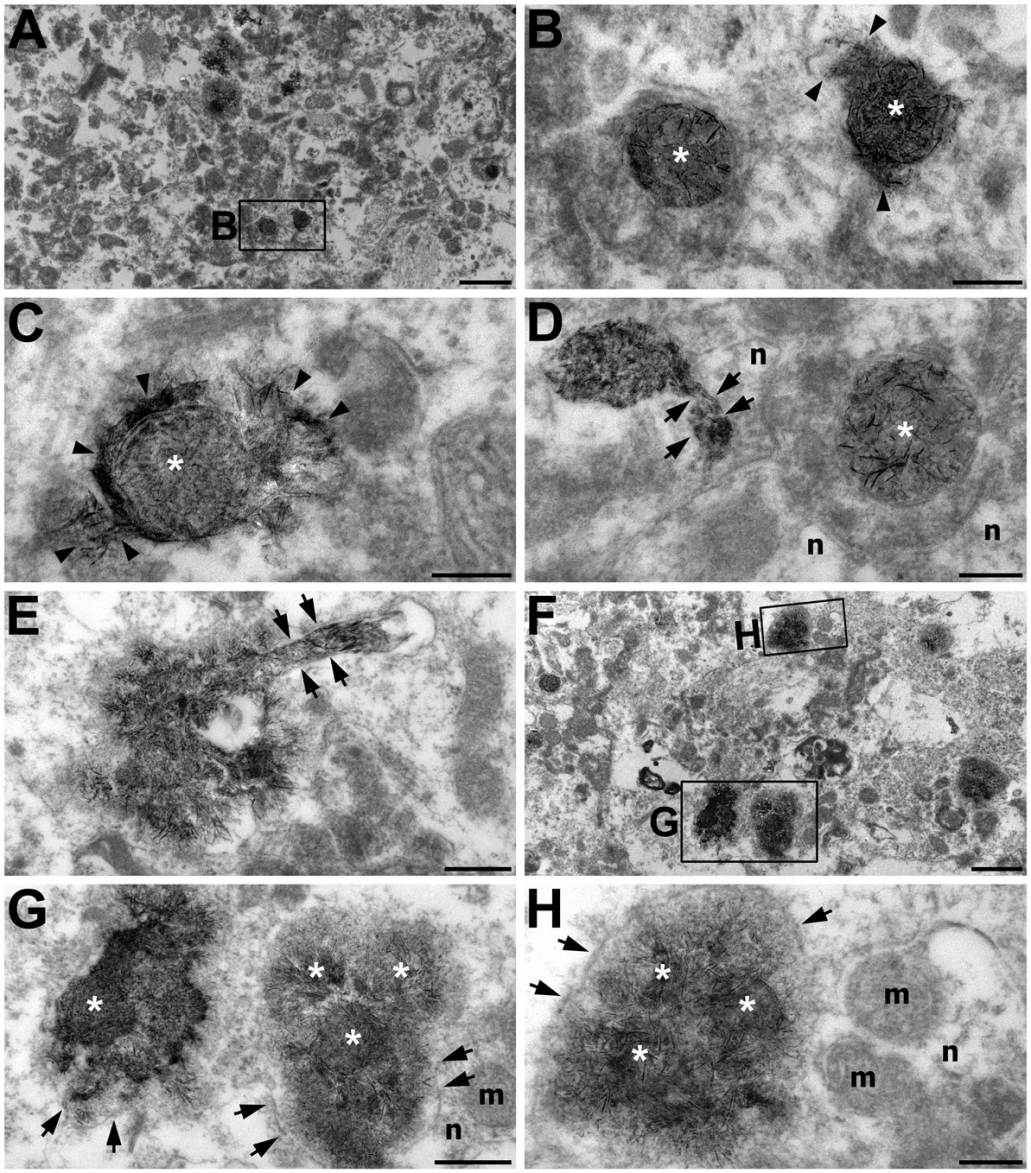
Electron photomicrographs of the CA1 hippocampal area at 14 days after reperfusion. The images were acquired using the osmium/potassium dichromate method. (A) Lower magnification view of the CA1 stratum radiatum showing that most areas were filled with darkly stained neurites and vacuoles of different sizes. The boxed area in A is enlarged in B. (B–E) Higher-magnification views of the CA1 stratum radiatum. Note that needle-shaped electron-dense crystals were localized within the mitochondria (white asterisks in B–D), or tended to spread beyond the mitochondria (arrowheads in B and C), and thereby along the dendrite-like profiles (arrows in D and E). Note that these dendritic profiles abutted the adjacent neurites (n in D). (F) Large irregular-shaped calcifying bodies were frequently observed in the CA1 stratum radiatum. (G, H) Higher magnification views of the boxed areas in F. These calcifying bodies contained central cores (white asterisks) of densely packed needle-like or rod-like calcium crystals and were surrounded by a membrane-like structure (arrows in G and H). Note that they were still in contact with neurites (n) containing non-calcifying mitochondria (m in G and H). Scale bars = 1 μm for A, F; 0.4 μm for E, G; 0.2 μm for B–D, H.

**Fig 5:**
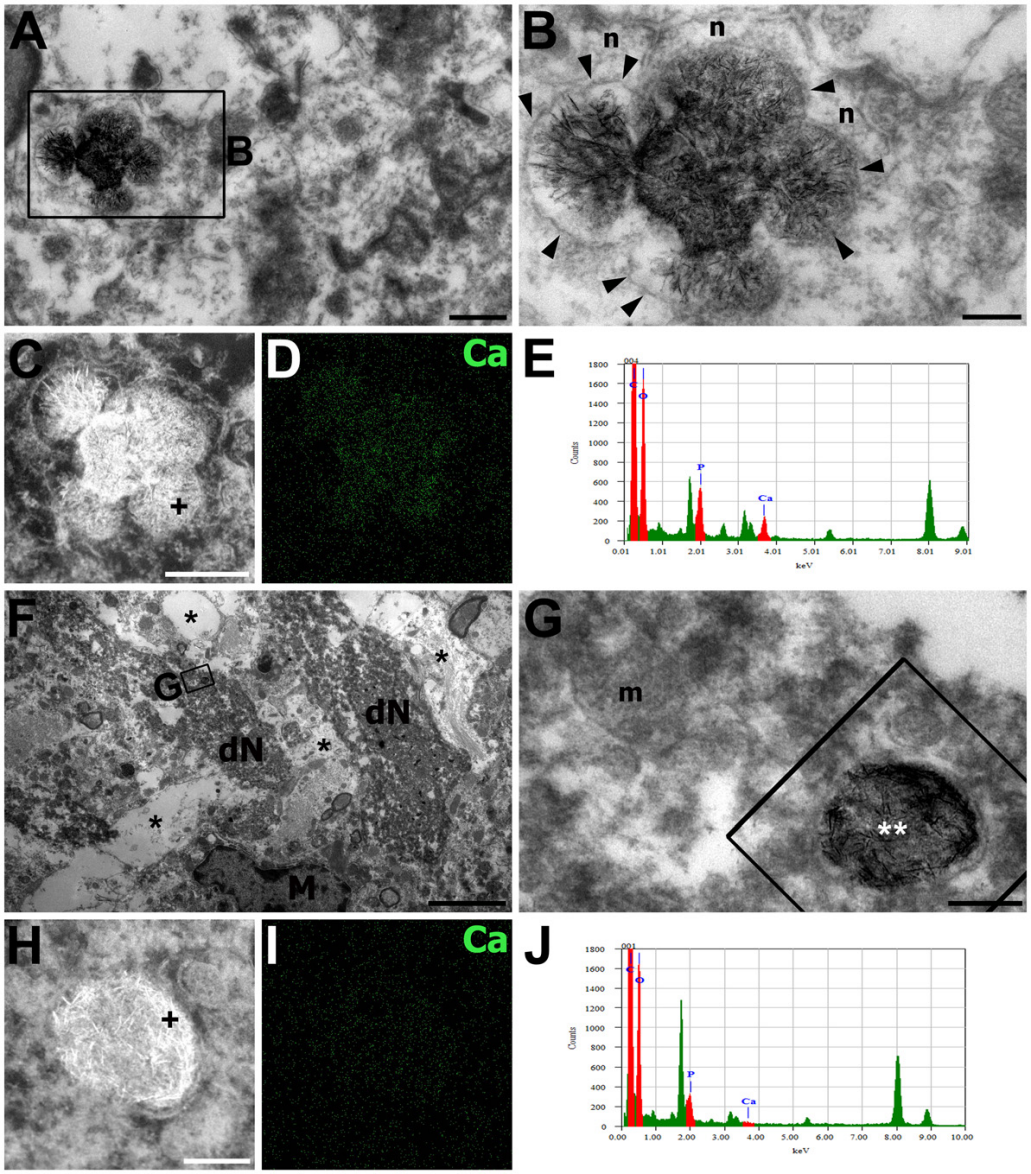
Electron photomicrographs of the CA1 hippocampal area at 14 days after reperfusion. The images were acquired using the osmium/potassium dichromate method. (A, B) A part of the CA1 stratum radiatum showing that the large calcifying bodies were filled with needle-shaped crystals and surrounded by membranous structures (arrowheads in b), which is suggestive of a conglomerate of adjacent calcifying neurites that were fused to each other. Note the calcifying body with adjacent neurites (n) abutting it. (B) Higher magnification view of the boxed area in A. (C–E) Dark-field images (C) and calcium maps (D) corresponding to the TEM images shown in B, respectively. Note the prominent signals for calcium in these structures. (E) X-ray spectrum analysis obtained from the selected point (cross) in C showing obvious peaks for calcium and phosphorous. (F) A part of the CA1 pyramidal cell layer showing homogeneously condensed electron-dense dead neurons (dN) and many empty spaces in place of the perikarya that underwent degeneration (asterisks) and activated microglia (M). (G) Higher magnification view of the boxed area in F. Note that the dark neuronal debris contained mitochondria (m) that had characteristic cristae and mitochondria-like profiles (white asterisk) filled with needle-shaped crystals. (H, I) Dark-field image (H) and calcium maps (I) corresponding to the TEM images shown in G. Note the absence of calcium signals in the electron-dense mitochondria. (J) X-ray spectrum analysis obtained from the selected point (cross) in H showing no significant peak for calcium. Scale bars = 4 μm for F; 0.5 μm for A, C, D; 0.2 μm for B, G–I.

Electron microscopy of the CA1 stratum radiatum at 14 days after reperfusion revealed that most areas were filled with darkly stained neurites and vacuoles of different sizes that were caused by neurite degeneration (Fig 4A). Degenerated dendrites were noticeably electron-dense and frequently contained mitochondria that were filled with needle-shaped calcium precipitates (Fig 4B–4D). The precipitates were not only localized within mitochondria, but also tended to spread beyond the mitochondria into the remainder of the dendroplasm (Fig 4B–4D) and along the dendrite-like profiles (Fig 4D and 4E). In addition to the calcified dendritic profiles, large irregular-shaped structures (<2 μm in dimension) were observed among the neuropils (Figs 4F–4H, 5A and 5B). These structures frequently contained central cores of densely packed needle-like or rod-like calcium crystals and were surrounded by a membrane-like structure (Figs 4G, 4H and 5B). These calcifying bodies were still in contact with degenerating or normal-appearing neurites that did not contain calcifying deposits within their mitochondria or cytoplasm. Based on these observations, the calcifying structures appeared to be a conglomerate of adjacent calcifying neurites that were fused to each other. FE-TEM with EDAX indicated that the needle-like crystal aggregates in these conglomerates were rich in calcium (Fig 5B–5D); moreover, calcium and phosphorous peaks were detected in these crystals (Fig 5E).

At 14 days after reperfusion, most of the neurons in the CA1 pyramidal cell layer were no longer discernible, but dark and markedly contracted neuronal remnants were observed among the microglial cells (Fig 5F). The remnants had neither nuclei nor recognizable cell organelles, except for mitochondria that still contained their characteristic cristae (Fig 5G). Some of the mitochondrial profiles were filled with needle-shaped crystals, although no prominent calcium signals were detected in these electron-dense mitochondrial profiles by FE-TEM with EDAX (Fig 5G–5J).

**Fig 6:**
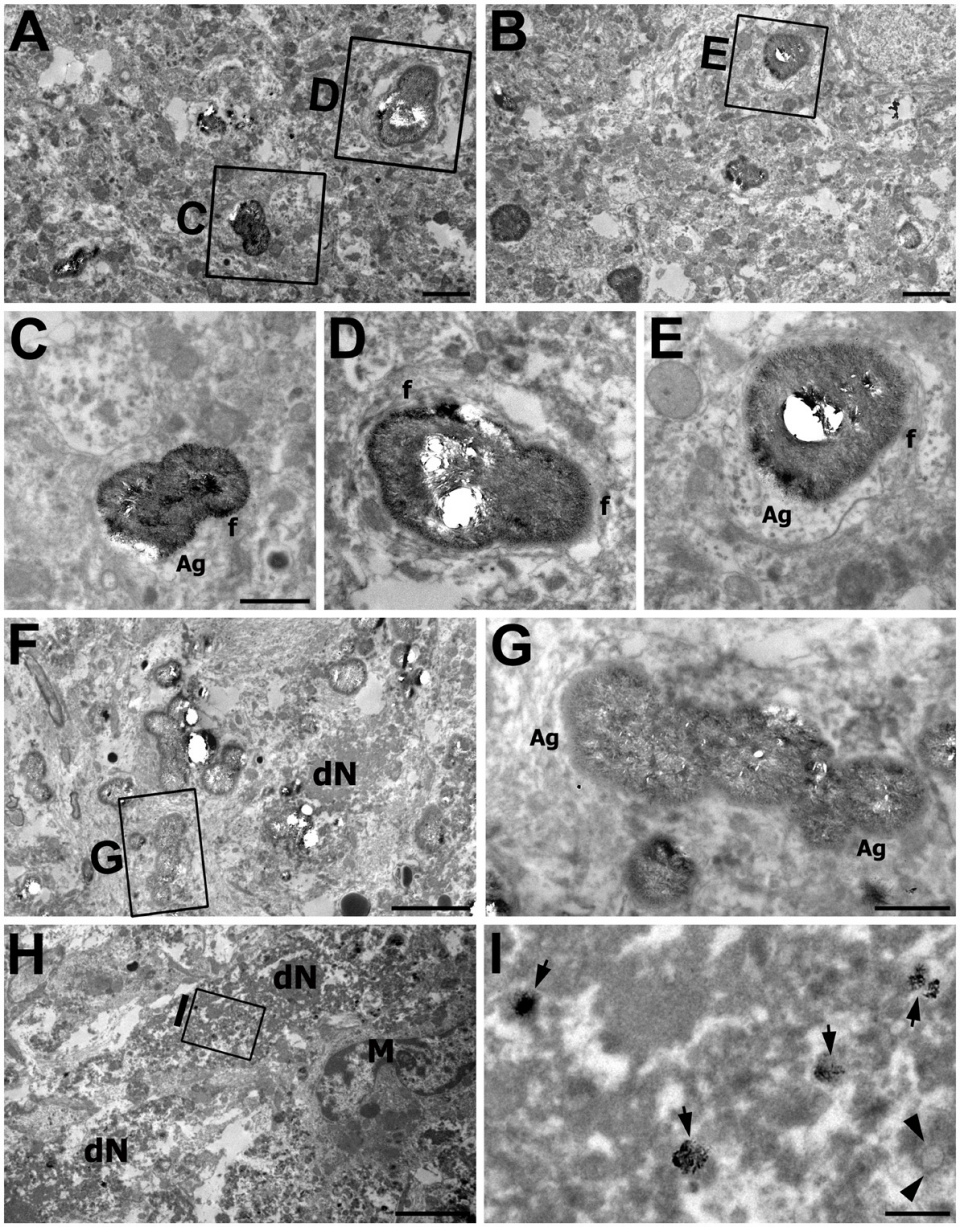
Electron photomicrographs of the CA1 hippocampal area at 28 days after reperfusion. The images were acquired using the osmium/potassium dichromate method. (A, B, F) Lower magnification views of the CA1 stratum radiatum showing that large calcifying bodies of variable sizes and shapes were frequently observed among the degenerated neurites. (C–E, G) Higher-magnification views of the boxed areas in A, B, and F, respectively. Needle-like calcium crystals were localized throughout the structures and on the peripheral outline of these calcifying bodies. Note that the calcifying bodies were surrounded by astrocytes (Ag) containing bundles of astroglial filaments (f). dN in F denotes dark cellular debris. (H) A part of the CA1 pyramidal cell layer. Dark cellular masses (dN) comprised of homogeneous fragments and microglial cells (M) were frequently observed in this layer. (I) Higher magnification view of the boxed area in H. Note the scattered mitochondrial profiles (arrows) that had densely packed needle-shaped crystals and one mitochondrial profile (arrowheads) that had no electron-dense material. Scale bars = 4 μm for F, H; 2 μm for A; 1 μm for C–E, G; 0.5 μm for B, I.

Sections from the CA1 stratum radiatum at 28 days after reperfusion revealed large calcifying bodies of variable sizes and shapes among the degenerated neurites (Fig 6A and 6B). Needle-shaped calcium crystals were present throughout the entire structure of the calcified bodies, and more intense calcium crystals were radially localized on their peripheral outline (Fig 6C–6E). These calcifying bodies were almost completely surrounded by astroglial processes that contained bundles of glial fibrils, and thus they appeared to be separated from the surrounding neurites. In addition, the calcifying bodies were often attached or fused to one another, thereby forming even larger calcified bodies that measured up to 4 μm (Fig 6F and 6G).

The CA1 pyramidal cell layer at 28 days after reperfusion was more compact with collapsed vacuoles compared to that at 14 days after reperfusion, and microglial cells and dark cellular debris that were composed of homogeneous fragments were observed (Fig 6H). At a higher magnification, electron-dense profiles, which appeared to be densely packed needle-shaped crystals within mitochondria-like structures, could be observed within these dark debris (Fig 6I).

## Discussion

In the present study, we performed a detailed ultrastructural analysis of the onset and spatiotemporal pattern of calcification in the ischemic hippocampus. Our results showed that intrahippocampal calcification was due to mineralization of the mitochondria within degenerated dendrites, with rapid extension of the mineralization eventually calcifying the entire dendroplasm, even in areas beyond the dendrites. Mitochondria sequester and buffer excess calcium ions under various pathologic conditions, including cerebral ischemia, in order to maintain cellular homeostasis [28, 29]. As the extramitochondrial Ca^2+^ concentration increases, both isolated and *in situ* brain mitochondria form electron-dense precipitates that are composed of both calcium and phosphate within the mitochondrial matrix, which provides safe crystallized storage for deleterious cations [30]. Thus, the finding that calcium deposits were evident within, but not beyond, the mitochondria within the first 3 days after reperfusion reinforces the idea that mitochondria may serve as the initial calcium buffering system after ischemic insults. To our knowledge, this is the first study to directly demonstrate the link between intramitochondrial calcification and extracellular calcification progression in response to ischemic insults.

Perhaps the most significant finding of the present study is that mineralized calcium deposits were produced in CA1 dendritic subfields rather than in the pyramidal cell layer after ischemia. At the light-microscopy level, very little alizarin red staining was detected in the CA1 pyramidal cell layer until day 28 after reperfusion, the latest time point examined, despite extensive cell death in this layer by 3 days after reperfusion. Instead, prominent alizarin red staining was detected in CA1 dendritic subfields, especially in the stratum radiatum, in the ischemic hippocampus starting 14 days after reperfusion, and the staining continued to increase for over 4 weeks. Using TEM with the osmium/potassium dichromate method and FE-TEM with EDAX, we detected calcium signals within the mitochondria of degenerating dendrites located in the ischemic CA1 stratum radiatum by 7 days after reperfusion. Furthermore, electron-dense mitochondria were observed within degenerating dendrites by 3 days after reperfusion, although we were unable to detect calcium signals in the dendrites, probably because the calcium signal was below the threshold for detection. Intramitochondrial calcification within these degenerated dendrites tended to lead to complete calcification of the entire dendrite, and even formed conglomerates consisting of adjacent calcifying neurites that were fused to each other at 14 days after reperfusion. Thus, our data indicate that the mitochondrial calcification of degenerating dendrites is a crucial initiating event for intrahippocampal calcification, which suggests that once an initial nucleation site, i.e., the dendritic mitochondria, is established, the progression of calcification occurs in the ischemic hippocampus.

Although calcium precipitation has been proposed as a protective buffer against free calcium ions [6, 7, 10, 12, 16], massive and progressive ectopic calcification is associated with progressive neurological deterioration [6–9]. These findings are in accordance with our results showing a close spatiotemporal relationship between calcium precipitation and neurodegeneration in the ischemic hippocampus. Therefore, we speculate that intrahippocampal calcification may lead to a debilitating loss of tissue function, making it a potential key factor in further neurodegenerative processes.

Compared to the CA1 stratum radiatum, no prominent calcification occurred over the perikarya or proximal dendrites of degenerating neurons even at 28 days after reperfusion. This finding is surprising since previous reports showed that extensive neuronal death occurs in the pyramidal cell layer of the CA1 hippocampal region starting within 2 to 3 days after transient forebrain ischemia and reaches its maximal effect within 1–2 weeks [17–19], and considering that calcification in the ischemic hippocampus has been ascribed to neuronal death [2, 6, 7, 9]. Here, TEM analysis revealed that at least two kinds of morphological changes occur in ischemic CA1 neurons, which is consistent with the results of previous studies [26, 31, 32]. Most pyramidal neurons revealed the characteristic features of necrotic cell death, that is, the disintegration of membrane integrity, dissolution of cytoplasmic organelles, and complete cellular disintegration, which were replaced by large vacuoles. However, no calcium deposits were detected in the vacuoles by 28 days after reperfusion. These results are in agreement with our *in vitro* results [33] and with previous findings indicating that no calcium deposition occurs in liquefactive or total necrosis [34]. In contrast, some neurons in the pyramidal cell layer did not display either swelling or membrane disruption, but instead became dark and condensed and retained their compact ultrastructure. Surprisingly, these dark neurons had some mitochondria that retained their cristae structures until the moment of neuronal disintegration, and these mitochondria were filled with densely packed needle-shaped crystals. However, their calcium levels were too low to be detected by electron probe microanalysis, and no further calcification beyond the mitochondria was detected in the perikarya of these dark neurons, even after the dark neurons became dark homogeneous masses composed of several fragments at 4 week after reperfusion.

These dark neurons share similar ultrastructural features with the more traditional “dark neurons,” which show dramatic compaction of the ultrastructural elements in the somatodendritic domain and die through non-necrotic, non-apoptotic cell death in *in vivo* ischemic conditions [26, 32, 35–39]. These “dark neurons” are observed even in undamaged environments either by a single electric shock or in the visibly intact hippocampus, in addition to in a necrotic or an excitotoxic environment such as those generated by ischemic insults or epilepsy [32, 37–40]. Recently, we reported that mineralized cells were detected in control hippocampal slice cultures but not in oxygen-glucose-deprived hippocampal cultures [33]. It is interesting that these *in vitro* mineralized cells closely resemble the dark neurons observed in the present study in terms of the presence of selective calcium deposition within, but not beyond, the mitochondria and because they both exhibit the ultrastructural features of non-necrotic, non-apoptotic cell death and retain their compact ultrastructure. In this regard, the dark neurons presented here and the mineralized neurons *in vitro* may correspond to the traditional “dark neurons,” and thus they may share similar histopathologies and modes of cell death.

It is also important to consider why the dendrites, rather than the somata, of degenerating neurons are prone to calcification in the ischemic hippocampus. Interestingly, it has been reported that calcium deposition preferentially occurs in degenerating dendrites after ischemic insults [41, 42] and after electrical stimulation or excitotoxic injury [13, 43, 44]. Moreover, dendritic and axonal morphological changes occur in advance of the neuronal somata changes in various neurodegenerative diseases [13, 43, 44]. In particular, Hasel, McKay [45] have demonstrated that the dendritic regions of cortical neurons, as compared to the somatic regions, are more susceptible to oxidative stress and excitotoxic injury, which is the major pathophysiology in the ischemic brain. Considering these previous findings and our observations, we speculate that dendritic mitochondria may be the first to respond to the calcium overload elicited by ischemic insults, thereby acting as sentinels that protect neurons in the ischemic brain. However, it is likely that degenerating dendrites are prone to calcification because of their differential sensitivity to calcium overload or because of other unknown mechanisms in the ischemic hippocampus. Furthermore, much remains to be learned about why calcification was only noted within, but not beyond, mitochondria in the somata of degenerating neurons; hence, further studies are needed to determine the precise mechanisms contributing to the differential calcification processes that occur in the dendrites and somata of degenerating neurons.

Another intriguing observation in the present study was that most of the calcifying bodies were closely associated with or completely engulfed by astrocytes; thus, they appeared to be separated from the surrounding neurites at 28 days after reperfusion, while they were in direct juxtaposition to adjacent calcifying or non-calcifying neurites by 14 days after reperfusion. These results are consistent with our previous *in vitro* and *in vivo* studies showing that the mineralized cells are closely associated with astrocytes [33, 41, 42] and with other studies showing that astrocytes may be involved in regulating the mineralization of neighboring degenerating cells [12, 42, 46]. Therefore, it is possible that astrocytes (1) participate in the phagocytosis of calcium deposits, (2) form glial barriers that surround the calcified structures and sequester them, and (3) regulate mineralization in the ischemic hippocampus. Additional studies are required to determine the precise role of astrocytes in the calcification process that occurs in response to brain insults.

It should also be noted that we used young (2-month-old) male rats, which have been used previously by many researchers in a variety of experimental studies. However, several studies have demonstrated that age and sex have robust effects on stroke outcome and that cortical neurons exhibit sex differences in response to ischemic cell death [47–50]. In this sense, additional studies are needed to characterize the differential effects of age and sex on the progression of microcalcification after ischemic insults.

## Conclusions

The present results indicate that intrahippocampal calcification after ischemic insults initially occurs in the dendritic mitochondria of degenerating neurons, with subsequent propagation of the mineralization occurring throughout the dendrites, which then coalesce to form larger mineral conglomerates of adjacent calcifying neurites. In contrast, no prominent calcification was observed in the CA1 pyramidal cell layer, even though what appeared to be calcifying mitochondria were noted in some of the degenerating neurons that became dark and condensed but retained their compact ultrastructure. Collectively, these results suggest that the calcified mitochondria in the dendrites, rather than in the somata, of degenerating neurons, may serve as a nidus for further calcium precipitation in the ischemic hippocampus.
